# Multi-Target Strategy to Uncover Unexpected Compounds in Rinse-Off and Leave-On Cosmetics

**DOI:** 10.3390/molecules26092504

**Published:** 2021-04-25

**Authors:** Maria Celeiro, Laura Rubio, Carmen Garcia-Jares, Marta Lores

**Affiliations:** Department of Analytical Chemistry, Nutrition and Food Science, Faculty of Chemistry, CRETUS Institute, Universidade de Santiago de Compostela, E-15782 Santiago de Compostela, Spain; laura.rubio.lareu@usc.es (L.R.); carmen.garcia.jares@usc.es (C.G.-J.); marta.lores@usc.es (M.L.)

**Keywords:** cosmetics analysis, banned compounds, polycyclic aromatic hydrocarbons, pesticides, nitrosamines, alkylphenol ethoxylates, miniaturized sample preparation, gas chromatography, liquid chromatography, mass spectrometry

## Abstract

The wide range and complexity of cosmetic formulations currently available on the market poses a challenge from an analytical point of view. In addition, during cosmetics manufacture, impurities coming from raw materials or formed by reaction of different organic compounds present in the formulation may be present. Their identification is mandatory to assure product quality and consumer health. In this work, micro-matrix solid-phase dispersion (μMSPD) is proposed as a multi-target sample preparation strategy to analyze a wide number of unexpected families of compounds including polycyclic aromatic hydrocarbons (PAHs), pesticides, plasticizers, nitrosamines, alkylphenols (APs), and alkylphenol ethoxylates (APEOs). Analytical determination was performed by gas chromatography-mass spectrometry (GC-MS) for the determination of 51 target compounds in a single run, whereas liquid chromatography tandem mass spectrometry (LC-MS/MS) was employed for the analysis of six APs and APEOs. Both methodologies were successfully validated in terms of linearity, accuracy, and precision in leave-on and rinse-off cosmetics. Limits of detection (LODs) were calculated in the low ng g^−1^, showing their suitability to determine trace levels of impurities and banned compounds with different chemical natures, providing useful tools to cosmetic control laboratories and companies.

## 1. Introduction

Cosmetics are complex matrices made up of large numbers and types of chemical compounds. In Europe, the Regulation EC 1223/2009 establishes rules to be complied with by any cosmetic product made available on the market, to ensure the functioning of the internal market and a high level of protection of human health [1]. In this way, the banned compounds and those restricted in terms of maximum permitted concentration are displayed in the different annexes (II and III, respectively) of the Regulation, which is in continuous review and update since its introduction [2]. In addition, to assure consumers health and to inform them about unsafe products, the European Commission has the Rapid Alert System (RAPEX), an early warning system for safety management. In the last 5 years, 419 products were withdrawn from the market due to the presence of forbidden compounds in their formulation [3].

The presence of banned substances is usually not due to the intentional addition by the manufacturer. They can proceed from impurities of allowed ingredients, or they can be formed by reaction of different organic compounds present in the formulation under particular setting conditions. A European Commission implementation decision [4] clearly states that the presence of traces of prohibited substances and impurities must remain at a level that is as low as reasonably achievable (ALARA) following Good Manufacturing Practice (GMP). When such presence is technically unavoidable, the cosmetics manufacturers are required to provide evidence of the technical unavoidability. That means they have to justify the presence of those traces by all necessary means. It is also recommended, especially in the case of non-threshold genotoxic and carcinogenic substances, that the cosmetic industry should keep improving its best practices in order to eliminate these substances in the finished cosmetic product. Among the unexpected families of compounds, polycyclic aromatic hydrocarbons (PAHs), pesticides, plasticizers, nitrosamines, alkylphenols (APs), or alkylphenol ethoxylates (APEOs), could be present in cosmetics.

Several cosmetic formulations, especially leave-on ones, are petrolatum or mineral oil-based to obtain a specific viscosity or to create a protector film on the skin (e.g., lip balms) [5]. However, permitted ingredients that come from petroleum distillation that might not be complete, may introduce PAHs in the final products through manufacturing processes. For their toxicity and carcinogenic properties, 16 PAHs have been catalogued as priority pollutants by the United States Environmental Protection Agency (EPA). The Regulation EC 1223/2009 listed PAHs as prohibited substances in cosmetic products and also set the content of benzo[a]pyrene (B[a]P) in raw material such as paraffin waxes and creosote oil to less than 0.005% *w*/*w* [1].

The presence of botanical-derived ingredients such as natural extracts or essential oils in cosmetics formulations is increasing, being considered as a positive quality by consumers [6]. However, pesticides can be used to improve the growth of the plants used as raw material and, although there is still no regulation of maximum residue levels (MRLs) for such plant-derived extracts, trace concentrations of pesticides could be detected in the finished products [7].

Plasticizers are employed in several cosmetic products such as nail polishes and hair sprays, and as solvents and perfume fixatives in many other products. These chemicals are linked to hormone disruption, which can affect development and fertility. However, whereas several of them such as dimethylphthalate (DMP) or diethylphthalate (DEP) are not restricted in their use as cosmetic ingredients, the majority of phthalates and bisphenol A (BPA) are completely forbidden. Despite this, the presence of banned plasticizers in cosmetics formulations have been reported, being mainly associated with a continuous migration from the plastic package. In fact, in products with plastic applicators to facilitate the transfer of the product to the area of application, it has been demonstrated that the level of phthalates provided by the applicator varies between 70–90% [8,9,10,11].

The presence of trace levels of nitrosamines in cosmetics is related with their presence in the raw materials, or they can be formed in their own formulation via reaction of nitrogen-containing compounds, especially secondary or tertiary amines, and nitrosating agents, such as nitrogen oxides or other allowed ingredients containing nitro groups, like the preservatives bronopol or bronidox. In this way, the Regulation establishes for allowed ingredients a maximum concentration of N-nitrosamine impurities of 50 µg kg^−1^, as well as its storage in nitrite-free containers [1].

APEOs are nonionic surfactants commonly used as emulsifiers and foaming agents. However, their biodegradation generates alkylphenols (APs), considered endocrine disruptors for their estrogenic effects. For this reason, APs are forbidden in the formulation of cosmetic products according to the Regulation EC 1223/2009, while some of the APEOs themselves, such as 4-nonylphenol ethoxylate (mono-, di, tri-) and their other forms marketed under the name of NP40 Alternative, continue to be used as emulsifying agents and surfactants in cosmetic products, although they must be controlled for their potential conversion into prohibited APs.

Taking into account the wide number of banned or unexpected compounds that could be present in the final cosmetic products, the cosmetic sector demands the development of reliable, fast, and easy-to-implement methodology to be able to determine a high number of compounds in a broad range of products in a single run.

The major drawback for the analysis of cosmetics is sample preparation since these matrices are complex and diverse. Most of the reported methodologies for their analysis are focused on specific families, that usually include a few target compounds or compounds with similar chemical properties [2,12,13]. Regarding sample preparation, solid–liquid (SLE) or liquid–liquid extraction (LLE) have been the most applied. However, they require high amounts of organic solvents and laborious experimental steps. Direct dilution (or ready-to-inject) is also one of the most employed procedures, but its main drawback is that it is a suitable option just for perfumes or simple matrices [14], but it is not adequate for complex matrices such as most of the currently marketed products, where a high number of compounds at different concentrations coexist. Besides, from a practical point of view, this approach can negatively affect the chromatographic system.

Therefore, the development of environmentally friendly sample preparation procedures that also imply an in situ clean-up step is a good and needed approach. In this way, matrix solid-phase dispersion (MSPD) has been proposed for the extraction of different families of cosmetic ingredients [15,16,17]. However, new trends in cosmetics sample preparation are moving towards the development of miniaturized procedures that comply with the green chemistry principles, and techniques such as solid-phase microextraction (SPME), ultrasound-assisted emulsification microextraction (USAEME), or stir bar sorptive dispersive microextraction (SBSDME) have been applied for the determination of fragrances, antioxidants, preservatives, PAHs, or MVOCs in cosmetics and personal care products [18,19,20,21,22,23]. In this way, a miniaturization of the classical MSPD (μMSPD), employing disposable low-cost material and a low organic solvent consumption (1 mL) is proposed as a suitable sample preparation technique. μMSPD has been reported for the extraction of allowed cosmetic ingredients such as fragrances, dyes, or UV filters [24,25,26,27]. However, to the best of our knowledge, this miniaturized technique has never been applied for the determination of a broad range of banned or unexpected compounds in cosmetics and personal care products.

Thus, the main goal of this work is the development of a multi-target strategy based on miniaturized sample preparation techniques followed by chromatographic analysis to cover a broad range of banned compounds with different chemical natures in leave-on and rinse-off cosmetics, providing useful tools to cosmetic control laboratories and production companies.

## 2. Results and Discussion

The target compounds, their CAS numbers, retention times, and quantification and identification ions (or MS/MS transitions for APEOs) are summarized in Table 1. A total number of 57 compounds including PAHs, pesticides, plasticizers, nitrosamines, APs, APEOs, oxidative dyes, and fragrances were considered. All of them, excluding APEOs that present different restriction levels (see footnotes in Table 1), are currently included in the Annex II “List of substances forbidden in cosmetics” of the Regulation EC No 1223/2009 [1].

### 2.1. Chromatographic Analysis

Since cosmetic formulations contain complex mixtures of several classes of different ingredients, the required multicomponent analysis becomes a challenge, making resolutive chromatography necessary [2,23]. Traditionally, cosmetics analysis methodology was based on liquid chromatography (LC) or gas chromatography (GC), offering both techniques robustness and a high-resolution power.

Choosing between GC or LC is mainly based on the physicochemical properties of the target analytes. In this work, since all the compounds (excluding APs and APEOs) were volatile or semivolatile, GC was proposed as the separation technique. For APs and APEOs, more polar and less volatile compounds, LC was selected as the most suitable option. It is important to note that few works regarding the determination of APs and APEOs in cosmetics and personal care products are reported in the literature since nonionic surfactants analysis represents a much higher level of complexity than other types of surfactants because several hundreds of individual substances may occur in a mixture [28]. This fact, together with the usual complexity of cosmetic matrices, make their analysis a challenge.

Chromatography with UV-based detectors has been the most employed technique for the determination of compounds present at high concentrations in the final products, such as preservatives, UV filters, or dyes [12]. However, to determine trace levels of banned compounds or impurities, the use of more selective detectors such as those based on mass spectrometry (MS) offer the selectivity and sensitivity required [29]. Therefore, in this work, GC-MS, working in the selected ion monitoring (SIM) mode, was selected for the analysis of PAHs, pesticides, plasticizers, nitrosamines, and the other target compounds (dyes, fragrances), making a total of 51 compounds.

On the other hand, to determine APEOs, fluorescence detection (FLD) has been the most selected and simple option. However, since the presence of APs is forbidden in cosmetics, LC coupled to tandem mass spectrometry (LC-MS/MS) working in the selection reaction monitoring (SRM) mode was selected as the determination technique for both the parent surfactants and their biodegradation products.

**Figure 1:**
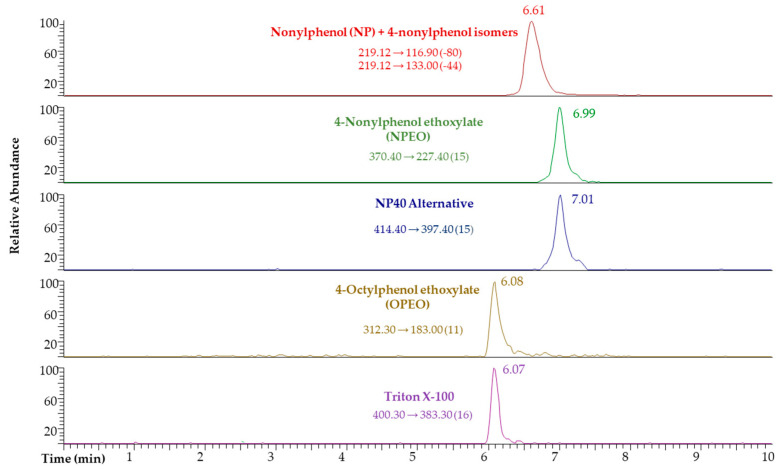
SRM reconstructed LC-MS/MS chromatogram for APs and APEOs (100 µg L^−1^ prepared in acetonitrile/water, 50:50, *v*/*v*).

In all cases, the chromatographic and determination conditions were optimized to obtain the highest separation and resolution efficiencies for an unequivocal identification of the target compounds. Conditions are summarized in Section 3.4. Figure 1 shows a SRM extracted (quantification transition) LC-MS/MS chromatogram for the considered APs and APEOs.

### 2.2. Sample Preparation Strategies

The latest advances in efficient and easy-to-implement cosmetic sample preparation methodology, as previously mentioned, have been focused on the development of miniaturized devices and procedures, reducing organic solvent consumption and sample amount, improving performance and, thus, lessening the environmental impact.

**Figure 2:**
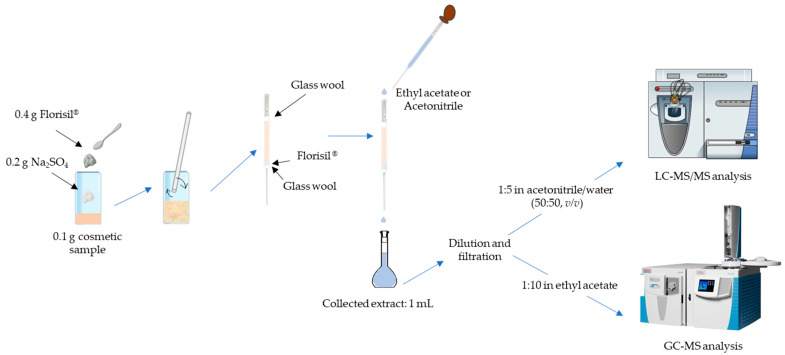
Schematic representation of the µMSPD experimental procedure.

In this way, μMSPD was selected as the extraction technique. Figure 2 shows the experimental procedure, which is further explained in Section 3.3.

### 2.3. Methods Performance

The proposed methodologies have been validated in terms of linearity, accuracy, and precision to show their suitability for the application to both leave-on and rinse-off cosmetics. In addition, limits of detection (LODs) were calculated. The methods performance parameters are summarized in Table 2 and Table 3 for µMSPD-GC-MS and µMSPD-LC-MS/MS, respectively.

#### 2.3.1. µMSPD-GC-MS

Calibration standards were prepared in ethyl acetate covering a concentration range between 10 and 2000 µg L^−1^ (plasticizers: 50–2000 µg L^−1^). The method exhibited a direct proportional relationship between the amount of each analyte and its chromatographic response, with coefficients of determination (R^2^) higher than 0.9910 in all cases (see Table 2).

Instrumental method precision was evaluated within a day (*n* = 3), and among days (*n* = 6) for all the calibration concentration levels. Relative standard deviation (RSD) values for 100 µg L^−1^ are also shown in Table 2. In all cases, the RSD values were lower than 9% and 11% for repeatability and reproducibility, respectively. To assess the accuracy of the proposed methodology, recovery studies were carried out employing two cosmetics samples: leave-on (moisturizing hand cream) and a rinse-off (shower gel) products. The study was performed by a sample addition of 100 µg g^−1^ for all compounds, for a validation range of 1 to 10 µg g^−1^, the actual concentrations in the injected dilutions, according to the potential presence of impurities in the finished cosmetic product under the ALARA principle. The spiked samples were extracted by µMSPD by triplicate and analyzed by GC-MS. As can be seen in Table 2, good accuracy and precision were achieved, with recovery values between 72–116%, and RSD values lower than 15% in all cases. Limits of detection (LODs) were calculated as the compound concentration giving a signal-to-noise ratio of three (S/N = 3) employing samples spiked with the target compounds. For the compounds that were detected in the whole procedure blanks (DIBP, DBP, and DEHP), LODs were calculated as the average amount of analyte giving a response that is the blank signal plus three times the standard deviation. Results are depicted in Table 2, and they were at the low ng g^−1^ level for all target compounds. Compared with other analytical methodologies based on GC-MS, the proposed µMSPD-GC-MS approach presents lower LODs (up to two orders of magnitude) than those reported for the analysis of PAHs or nitrosamines in cosmetics employing solid–liquid extraction-GC-MS/MS [30,31]. Other advantages of the proposed µMSPD procedure are that only 1 mL of organic solvent (ethyl acetate) is required, and the inclusion of an in situ clean-up step allows for a high fractionation degree, obtaining clean extracts that can be directly injected without further preparation steps.

#### 2.3.2. µMSPD-LC-MS/MS

Calibration standards were prepared in acetonitrile/water (50:50, *v*/*v*) covering a range between 2 and 10,000 µg L^−1^. The specific linear range for each target compound is shown in Table 3**.** The method exhibited a direct proportional relationship between the amount of each analyte and its chromatographic response, with coefficients of determination (R^2^) higher than 0.9974 in all cases.

Instrumental method precision was evaluated within a day (n = 3) and among days (n = 6) for all the calibration concentration levels. Relative standard deviation (RSD) values for 100 µg L^−1^ are also shown in Table 3. In all cases, the RSD values were lower than 10% for repeatability and reproducibility.

It is well-known that matrix effect, the ionization suppression or enhancement of the analyte of interest by other compounds present in the sample, becomes a major problem for the analysis of complex samples, such as cosmetics, using LC-MS/MS. Matrix effect was assessed for each of the target APs and APEOs by comparing the slopes obtained for external calibration and those obtained employing matrix-matched calibration. Results are shown in Table S2. For the rinse-off sample (shower gel), values resulting from dividing the slope of both curves were 1.0 ± 0.2, demonstrating that no matrix effects exist. However, for OPEO and Triton X-100, positive and negative matrix effects were observed, respectively, for the leave-on (moisturizing cream) cosmetic. Therefore, for these two compounds, matrix-matched calibration is recommended to analyze leave-on samples, whereas for the rinse-off ones, external calibration employing standards prepared in acetonitrile/water (50:50, *v*/*v*) is a suitable option. Recovery studies were carried out employing both samples spiked at 20 µg g^−1^ for all APs and APEOs. The spiked samples were extracted by µMSPD by triplicate and analyzed by LC-MS/MS. As can be seen in Table 3, recovery values ranged between 83 and 106%, with RSD values lower than 11% in all cases. LODs were also calculated as the compound concentration giving a signal-to-noise ratio of three (S/N = 3) since none of the target compounds were detected in the procedure blanks. Obtained values ranged between 0.09 and 1.30 ng g^−1^ being well below, by up to 2 orders of magnitude, those reported in the literature employing ultrasound assisted extraction (UAE)-LC-FLD or solid–liquid extraction followed by LC-MS/MS [32,33].

## 3. Materials and Methods

### 3.1. Reagents and Materials

The 57 target compounds, their CAS numbers, retention times, and MS ions or MS/MS transitions are summarized in Table 1. Ethyl acetate was supplied by Sigma-Aldrich Chemie GmbH (Steinheim, Germany), acetonitrile (MS grade) and acetone were provided by Fluka Analytical (Steinheim, Germany). Water (MS grade) was purchased from Scharlab (Barcelona, Spain). Anhydrous sodium sulfate, Na_2_SO_4_ (99%) was obtained from Panreac (Barcelona, Spain). Florisil^®^ (60–100 µm mesh) and glass wool were purchased form Supelco Analytical (Bellefonte, PA, USA). Individual stock solutions of each target analyte were prepared in ethyl acetate or methanol for APs and APEOs. Further dilutions and mixtures were prepared in ethyl acetate or acetonitrile/water (50:50, *v*/*v*) for GC-MS and LC-MS/MS analysis, respectively, and in acetone for spike solutions. All solutions were stored at −20 °C. All reagents were of analytical grade.

Since the target compounds include plasticizers (mainly phthalates that are ubiquitous compounds), to avoid contamination during the experimental procedure, all the plastic material was substituted, as far as possible, by glass or metallic material that was kept at 230 °C for at least 12 h before its use.

### 3.2. Cosmetic Samples

Two cosmetic samples, a moisturizing hand cream (leave-on) and a shower gel (rinse-off) were selected to validate the proposed methodology. Both samples were selected because they were labeled as fragrance, parabens, and silicone free (leave-on sample composition: aqua (water), paraffinum liquidum, glycerin, glyceryl stearate se, bis-diglyceryl polyacryladipate-2, stearic acid, cetearyl alcohol, urea, potassium stearate, creatine, 1,2-hexanediol, caprylyl glycol, tropolone; rinse-off sample composition: Aqua, sodium laureth sulfate, glycerin, cocamidopropyl betaine, sodium chloride, coco-glucoside, parfum, sodium lactate, lactic acid, sodium benzoate). They were kept in their original containers and protected from light at room temperature until their use.

### 3.3. Micro-MSPD Procedure

The µMSPD procedure was adapted from that previously developed by the authors for the extraction of fragrances, UV filters, or preservatives from cosmetic and personal care products [9,26,27]. Briefly, 0.1 g of cosmetic samples were weighted into a 10 mL glass vial. Then, the sample was gently blended with 0.2 g of Na_2_SO_4_ (drying agent) and 0.4 g of Florisil^®^ (dispersant) in the vial, using a glass rod, until a homogeneous mixture was obtained. The mixture was then transferred into a glass Pasteur pipette (150 mm), containing a small amount of glass wool at the bottom, and about 0.1 g of Florisil^®^ (to obtain a high fractionation degree and an in situ clean-up step). Finally, a small amount of glass wool was placed on the top to compress the mixture. Elution with ethyl acetate (GC-MS analysis) or acetonitrile (LC-MS/MS analysis) was carried out by gravity flow, collecting 1 mL of extract in a volumetric flask. The obtained extracts were diluted 1:10 (*v*/*v*) in ethyl acetate (GC-MS analysis) and 1:5 (*v*/*v*) in acetonitrile/water (50:50, *v*/*v*) (LC-MS/MS analysis), filtered through 0.22 µm polytetrafluoroethylene (PTFE) filters, and analyzed by GC-MS or LC-MS/MS.

For the recovery studies, the sample was spiked with 10 µL of the corresponding acetonic solution containing the target compounds to achieve the desired final concentration of them and submitted to the same process described above. Blanks procedures were daily performed to evaluate the presence of the target compounds (mainly plasticizers) during the experimental process. Figure 2 illustrates the described µMSPD methodology.

### 3.4. GC-MS Analysis

The GC-MS analysis was performed using an Agilent 7890A coupled to an Agilent 5975C inert mass spectrometer detector (MSD) with triple-axis detector and an Agilent 7693 autosampler from Agilent Technologies (Palo Alto, CA, USA). The separation was achieved employing a ZB-Semivolatiles (30m × 0.25 mm i.d., 0.25 µm film thickness) column obtained from Phenomenex (Torrance, CA, USA), with a chromatographic ramp that applies 50 °C (held 3 min) to 200 °C at 4 °C min^−1^, and a final ramp to 290 °C at 20 °C min^−1^ (held 3 min). The total run time was 50 min. Helium (purity 99.999%) was employed as a carrier gas at a constant flow of 1.0 mL min^−1^. The sample volume was 1 µL, and the injector temperature was set at 270 °C. The MSD was operated in the electron impact (EI) ionization positive mode (+70 eV). The temperature of the ion source was 150 °C and the transfer line temperature was set at 290 °C. Selected ion monitoring (SIM) acquisition mode was employed, monitoring 2 or 3 mass/charge (*m*/*z)* fragments for each compound for an unequivocal identification.

### 3.5. LC-MS/MS Analysis

The LC-MS/MS analyses were performed employing a Thermo Fisher Scientific (San José, CA, USA) instrument based on a TSQ Quantum Ultra^TM^ triple quadrupole mass spectrometer equipped with a HESI-II (heated electrospray ionization), and an Accela Open autosampler with a 20 µL loop. The chromatographic separation was achieved on a Kinetex C18 EVO column (100 × 2.1 mm, 2.6 µm, 100 Å), obtained from Phenomenex. The temperature of the column was set at 30 °C. The mobile phase consisted of water (A) and ACN (B), both with 5 mM of NH_4_OH, since basic pH containing NH_4_^+^ ions favors the formation of [APEO+NH_4_]^+^ adducts, that present a high fragmentation grade in MS/MS.

The elution gradient started with 55% of B, it was increased to 100% of B in 7 min and kept constant for 1 min. Finally, initial conditions were reached in 3 min. The injection volume was 10 µL and the mobile phase flow-rate was 0.2 mL min^−1^. The total run for each injection was 25 min. The spray voltage was 3000 V, the vaporization and capillary temperatures were set at 300 and 350 °C, respectively. Pressure sheath, sweep, and auxiliary gas were kept at 28, 2, and 5 au (arbitrary units), respectively. The mass spectrometer and the HESI source were working simultaneously in the positive and negative mode, monitoring different MS/MS transitions for each compound for an unequivocal identification. Quantification MS/MS transitions are shown in Table 1 and confirmation ones are summarized in Table S1.

## 4. Conclusions

µMSPD has been demonstrated to be a suitable sample preparation procedure to analyze traces of prohibited substances described by the EU Cosmetics Regulation as stemming from impurities of natural or synthetic ingredients, the manufacturing process, storage, or migration from packaging, that are technically unavoidable in good manufacturing practice and, thus, whose presence is non-intended. The proposed method enables the extraction of a wide number of impurities and banned compounds such as PAHs, pesticides, plasticizers, nitrosamines, Aps, and APEOs in leave-on and rinse-off cosmetics. The use of GC-MS and LC-MS/MS as determination techniques provide the required selectivity and analyte sensitivity to detect trace levels of the target compounds. The proposed methodologies were successfully validated in terms of linearity and precision. Recovery studies were also performed, being quantitative in both cosmetic matrices. LODs were at the low ng g^−1^ level for all compounds. Therefore, the combination of µMSPD with chromatographic-mass spectrometric techniques appears to be a very suitable tool for cosmetic control laboratories and manufacturers to determine trace levels of the target compounds in the final products in order to assure cosmetics quality, legal compliance, and, above all, consumer and user health and safety.

## Figures and Tables

**Table 1:** Studied compounds. CAS; retention time; and quantification and identification ions or MS/MS transitions.

Compounds	Acronym	CAS	Ret. Time (min)	Quantification and Identification Ions (Relative Abundance)
**PAHs**				
Naphthalene	NAP	91-20-3	16.11	128 (100), 129 (11), 127 (11)
Acenaphthylene	ACY	208-96-8	24.96	152 (100), 153 (15), 151 (14)
Acenaphthene	ACE	83-32-9	26.00	153 (100), 154 (83), 152 (51)
Fluorene	FLU	83-73-7	29.00	166 (100), 165 (84), 167 (14)
Phenanthrene	PHEN	85-01-8	34.41	178 (100), 176 (20), 179 (15)
Anthracene	ANC	120-12-7	34.70	178 (100), 179 (16), 176 (14)
Fluoranthene	FLA	206-44-0	41.26	202 (100), 203 (17), 200 (15)
Pyrene	PYR	129-00-0	42.63	202 (100), 203 (17), 100 (15)
Benzo[a]anthracene	B[a]A	56-55-3	46.49	228 (100), 226 (28), 229 (20)
Chrysene	CHY	218-01-9	46.55	228 (100), 226 (27), 229 (20)
Benzo[b]fluoranthene	B[b]F	205-99-0	48.41	252 (100), 253 (22), 250 (18)
Benzo[k]fluoranthene	B[k]F	207-08-9	48.47	252 (100), 253 (21), 250 (21)
Benzo[a]pyrene	B[a]P	50-32-8	49.06	252 (100), 253 (21), 250 (27)
**Pesticides**				
Monocrotophos	MNC	6923-22-4	32.23	127 (100), 97 (21), 192 (15)
Chlorpyrifos-methyl	CPM	5598-13-0	36.84	286 (100), 125 (95), 288 (78)
Simazine	SIM	122-34-9	33.48	201 (100), 186 (62), 173 (47)
Propazine	PRZ	139-40-2	33.92	214 (100), 229 (53), 172 (47)
Chlorpropham	CP	101-21-3	31.27	127 (100), 213 (30), 171 (21)
Kresoxim-methyl	KRM	143390-89-0	44.31	116 (100), 131 (56), 206 (52)
Iprodione	IPR	36734-19-7	46.38	314 (100), 187 (70), 70 (50)
Myclobutanil	MYC	88671-89-0	44.10	179 (100), 152 (40), 181 (32)
Tebuconazole	TBC	107534-96-3	45.95	125 (100), 250 (50), 83 (49)
4,4′DDT	DDT	50-29-3	45.70	235 (100), 237 (65), 165 (43)
Carbaryl	CAR	63-25-2	37.48	144 (100), 115 (56), 116 (38)
Alachlor	ALA	15972-60-8	37.26	160 (100), 188 (90), 237 (50)
Dieldrin	DIE	60-57-1	43.88	79 (100), 263 (20), 277 (15)
**Plasticizers**				
Diisobutylphthalate	DIBP	84-69-5	36.55	149 (100, 57 (15), 223 (6)
Dibutylphthalate	DBP	84-74-2	38.90	149 (100), 150 (9), 223 (5)
Dimethoxyethylphthalate	DMEP	117-82-8	39.76	59 (100), 104 (18), 149 (29)
Diisopentylphthalate	DIPP	605-50-5	41.50	149 (100), 71 (28), 237 (10)
Dipentylphthalate	DPP	131-18-0	46.88	149 (100), 71 (16), 237 (6)
Benzylbutylphthalate	BBP	85-68-7	46.67	149 (100), 91 (53), 206 (24)
Di-(2-ethylhexyl)phthalate	DEHP	117-81-7	46.88	149 (100), 167 (30), 279 (10)
Bisphenol A	BPA	80-05-7	43.91	213 (100), 119 (19), 228 (25)
**Nitrosamines**				
*N*-nitrosodiethylamine	NDEA	55-18-5	5.96	102 (100), 44 (76), 56 (54)
*N*-nitrosopyrrolidine	NPYR	930-55-2	11.81	100 (100), 41 (69), 68 (13)
*N*-nitrosodipropylamine	NDPA	621-64-7	12.03	130 (100), 43 (117), 70 (66)
*N*-nitrosomorpholine	NMOR	59-89-2	12.04	116 (100), 56 (120), 86 (42)
*N*-nitrosopiperidine	NPIP	100-75-4	13.36	114 (100), 42 (117), 55 (61)
*N*-nitrosodibutylamine	NDBA	924-16-3	18.97	84 (100), 116 (31), 158 (15)
*N*-nitrosodiphenylamine	NDPhA	86-30-6	30.16	168 (100), 77 (16), 167 (57)
*N*-nitroso-*N*-methylaniline	NMA	614-00-6	11.87	106 (100), 77 (50), 107 (44)
*N*-nitroso-*N*-ethylaniline	NEA	612-64-6	14.27	77 (100), 120 (71), 106 (46)
*N*-nitrosodibenzylamine	NDBzA	5336-53-8	38.02	91 (100), 226 (11), 65 (14)
**Other compounds: oxidative dyes and fragrances**				
1,4-dihydroxybenzene	Hydroquinone	123-39-1	6.86	110 (100), 55 (18), 81 (31)
2-naphthol	2-NAP	135-19-3	27.24	144 (100), 115 (71), 116 (24)
Versalide	ATTN	88-29-9	34.81	243 (100), 244 (18), 258 (27)
Musk ambrette	MA	83-66-9	35.48	253 (100), 254 (13), 268 (35)
Musk moskene	MM	116-66-5	36.80	263 (100), 264 (20), 278 (9)
Musk tibetene	MT	145-39-1	37.89	251 (100), 43 (33), 266 (28)
4-(4-Hydroxy-4-methylpentyl)-3-cyclohexene-1-carboxaldehyde	Lyral^®^	31906-75-4	31.43	136 (100), 79 (74), 93 (78)
**APs and APEOs**	**Acronym**	**CAS**	**Ret. Time(min)**	**MS/MS Transitions (Collision Energy, eV) ^a^**
Nonylphenol	NP	25154-52-3	6.61	219.12 → 133.0 (−44)
4-nonylphenol isomers	4NP	84852-15-3	6.61	219.1 → 116.9 (−80)
4-nonylphenol ethoxylate ^b^	NPEO	68412-54-4	6.99	370.40 → 227.40 (15)
4-octylphenol ethoxylate ^c^	OPEO	26636-32-8	6.08	312.30 → 183.00 (11)
NP40 Alternative ^b^	NP40	9016-45-9	7.01	414.10 → 397.40 (15)
Triton X-100 ^c^	TX-100	9002-93-1	6.30	400.30 → 383.30 (16)

^a^ Quantification MS/MS transition. Confirmation MS/MS transitions are included in Table S1. ^b^ Used as surfactant (cleansing and emulsifying). ^c^ Not regulated according to Regulation EC No 1223/2009.

**Table 2:** µMSPD-GC-MS performance. Coefficient of determination (R^2^), precision, accuracy, and limits of detection (LOD).

Compounds	R^2^	Precision		Recovery, %		LOD (ng g^−1^) ^a^
		Intra-Day, RSD, %	Inter-Day, RSD, %	Leave-On	Rinse-Off	
**PAHs**						
NAP	0.9993	1.2	10	109 ± 1	108 ± 10	1.0
ACY	0.9980	4.3	9.3	107 ± 5	97 ± 11	2.0
ACE	0.9995	5.9	8.8	116 ± 6	98 ± 9	4.0
FLU	0.9991	2.3	7.5	117 ± 1	99 ± 10	6.2
PHEN	0.9987	6.3	4.9	114 ± 2	97 ± 9	3.8
ANC	0.9998	7.2	10	119 ± 1	96 ± 11	3.9
FLA	9.9989	7.4	11	102 ± 1	98 ± 8	3.5
PYR	0.9986	3.4	6.4	99 ± 13	98 ± 10	2.5
B[a]A	0.9988	3.5	8.7	101 ± 9	102 ± 11	1.9
CHY	0.9990	6.0	11	98 ± 13	95 ± 9	2.0
B[b]F	0.9974	3.2	13	94 ± 7	106 ± 14	10
B[k]F	0.9972	3.5	6.7	94 ± 8	98 ± 16	10
B[a]P	0.9984	1.8	4.3	89 ± 9	101 ± 9	12
**Pesticides**						
MNC	0.9978	1.2	3.2	n.c.	99 ± 3	250
CPM	0.9972	3.2	4.5	106 ± 8	105 ± 10	5.9
SIM	0.9910	4.1	6.3	108 ± 7	107 ± 1	150
PRZ	0.9971	2.1	5.8	100 ± 3	98 ± 2	70
CP	0.9932	5.4	10	89 ± 3	100 ± 1	90
KRM	0.9978	1.0	4.2	104 ± 10	112 ± 1	15
IPR	0.9994	3.6	10	87 ± 7	120 ± 4	50
MYC	0.9968	1.3	2.6	102 ± 6	120 ± 1	7.5
TBC	0.9992	1.2	5.6	113 ± 16	100 ± 2	9.0
DDT	0.9996	3.2	4.6	81 ± 1	99 ± 9	7.3
CAR	0.9987	5.1	8.2	87 ± 10	112 ± 7	87
ALA	0.9967	8.9	10	106 ± 7	117 ± 2	15
DIE	0.9923	5.1	9.6	82 ± 4	107 ± 1	15
**Plasticizers**						
DIBP	0.9992	5.8	7.4	102 ± 7	88 ± 3	10
DBP	0.9990	6.2	10	91 ± 4	85 ± 3	7.5
DMEP	0.9991	2.9	7.5	89 ± 6	88 ± 2	37
DIPP	0.9982	6.3	11	77 ± 2	98 ± 1	10
DPP	0.9992	5.9	10	90 ± 3	86 ± 2	6.4
BBP	0.9976	8.8	10	80 ± 5	105 ± 15	34
DEHP	0.9998	5.1	7.8	78 ± 3	99 ± 1	15
BPA	0.9953	1.5	5.6	n.c.	110 ± 2	n.c.
**Nitrosamines**						
NDEA	0.9961	1.6	3.8	99 ± 2	103 ± 8	26
NPYR	0.9949	1.5	2.5	119 ± 2	118 ± 2	60
NDPA	0.9943	0.7	3.5	115 ± 1	106 ± 8	12
NMOR	0.9979	2.5	5.6	101 ± 3	101 ± 6	75
NPIP	0.9979	1.5	6.3	112 ± 2	115 ± 8	42
NDBA	0.9972	2.1	5.2	116 ± 2	118 ± 8	90
NDPhA	0.9998	2.3	15	116 ± 3	118 ± 2	12
NMA	0.9973	1.4	7.6	107 ± 12	102 ± 10	40
NEA	0.9928	2.3	3.6	90 ± 4	100 ± 3	150
NDBzA	0.9976	3.8	9.2	109 ± 10	101 ± 4	86
**Other compounds: oxidative dyes and fragrances**						
Hydroquinone	0.9989	3.6	5.2	71 ± 9	89 ± 10	200
2-NAP	0.9980	7.1	9.0	106 ± 7	110 ± 12	71
ATTN	0.9996	4.3	6.5	117 ± 2	94 ± 1	4.1
MA	0.9965	3.6	4.4	101 ± 3	83 ± 2	10
MM	0.9933	2.6	4.3	107 ± 6	77 ± 9	12
MT	0.9964	4.2	4.0	89 ± 10	83 ± 2	8.8
Lyral^®^	0.9937	4.6	5.5	114 ± 2	97 ± 1	20

^a^ LODs were calculated for the leave-on cosmetic sample. n.c. Not calculated since there was matrix interference.

**Table 3:** µMSPD-LC-MS/MS performance. Coefficient of determination (R^2^), precision, accuracy, and limits of detection (LOD).

APs and APEOs	Linearity		Precision		Recovery, %		LOD (ng g^−1^)	
	Linear Range, (µg L^−1^)	R^2^	Intra-Day, RSD, %	Inter-Day, RSD, %	Leave-On	Rinse-Off	Leave-On	Rinse-Off
NP + 4 NP	2–10,000	0.9998	2.3	8.4	90 ± 5	83 ± 7	0.11	0.09
NPEO	10–10,000	0.9992	5.6	9.2	106 ± 7	88 ± 10	1.12	0.36
OPEO	10–10,000	0.9974	4.4	5.0	92 ± 4	94 ± 11	1.30	0.26
NP40	10–10,000	0.9998	8.7	9.1	91 ± 3	90 ± 3	0.22	0.37
TX-100	20–10,000	0.9997	9.9	10	100 ± 2	96 ± 2	1.14	0.65

## Supplementary Materials

Supplementary materials are available online. Table S1. Confirmation MS/MS transitions for the APs and APEOs determination by µMSPD-LC-MS/MS, Table S2. Matrix effects assessment for APs and APEOs analysis by µMSPD-LC-MS/MS.
