# Supplementary material for: Multi-Target Strategy to Uncover Unexpected Compounds in Rinse-Off and Leave-On Cosmetics
Source: Molecules. 2021 Apr 25;26(9):2504. doi: 10.3390/molecules26092504 (PMC8123312; doi:10.3390/molecules26092504)
Supplement: Supplementary file 1 [file molecules-26-02504-s001.zip › molecules-1197817-supplementary.pdf]

Supplementary material

Multi-target strategy to uncover unexpected compounds in rinse-off and leave-on cosmetics

Maria Celeiro*, Laura Rubio, Carmen Garcia-Jares and Marta Lores

CRETUS Institute, Department of Analytical Chemistry, Nutrition and Food Science, Faculty of Chemistry, Universidade de Santiago de Compostela, E-15782, Santiago de Compostela, Spain; maria.celeiro.montero@usc.es (M.C); laura.rubio.lareu@usc.es (L.R); carmen.garcia.jares@usc.es (C.G-J) marta.lores@usc.es (M.L)

* Correspondence: maria.celeiro.montero@usc.es (MC); Tel.: (+34-881814464)

Content:

Table S1. Confirmation MS/MS transitions for the determination of APs and APEOs by μ-MSPD-LC-MS/MS.

Table S2. Matrix effects assessment for APs and APEOs analysis by μ-MSPD-LC-MS/MS.

Table S1. Confirmation MS/MS transitions for the determination of APs and APEOs by μ-MSPD-LC-MS/MS.

APs and APEOs	Ionization mode	Fragments	Precursor ion	Product ions	Collision energy (eV)
**NP + 4-NP isomers**	-	NP + 4-NP isomers	219.1	116.9	-80
				133.0	-44
			219.1	117.0	-86
				133.0	-44
				147.0	-34
**NPEO**	+	NPEO2	326.4	183.0	13
		NPEO3	370.4	227.4	15
**OPEO**	+	OPEO2	312.3	183.0	11
		OPEO3	356.3	227.0	12
**NP40**	+	NPEO4	414.1	397.4	15
		NPEO5	458.4	441.4	19
		NPEO6	502.5	485.5	20
		NPEO7	546.5	529.5	23
		NPEO8	590.5	573.5	22
		NPEO9	634.5	617.5	25
		NPEO10	678.4	661.50	28
		NPEO11	722.6	705.6	28

		NPEO12	766.6	749.6	29
		NPEO13	810.6	793.6	31
		NPEO14	854.6	837.6	33
		NPEO15	898.6	881.6	34
		NPEO16	942.6	925.6	36
		NPEO17	986.6	969.6	36
		NPEO18	1030.7	1013.7	37
		NPEO19	1074.7	1057.7	38
		NPEO20	1118.7	1101.7	40
**Triton X-100**	+	OPEO4	400.3	383.3	16
		OPEO5	444.4	427.4	18
		OPEO6	488.4	471.4	21
		OPEO7	532.4	515.4	24
		OPEO8	576.4	559.4	25
		OPEO9	620.4	603.4	27
		OPEO10	664.4	647.4	28
		OPEO11	708.5	691.5	29
		OPEO12	752.5	735.5	30
		OPEO13	796.5	779.5	31
		OPEO14	840.6	823.6	31
		OPEO15	884.6	867.6	33
		OPEO16	928.6	911.6	34
		OPEO17	972.6	955.6	35
		OPEO18	1016.70	999.7	36
		OPEO19	1060.7	1043.7	37
		OPEO20	1104.80	1087.80	38

Table S2. Matrix effects assessment for APs and APEOs analysis by µ-MSPD-LC-MS/MS.

APs and APEOs	External calibration	Matrix-matched		SMM/SEC[a]	
		Leave-on	Rinse-off	Leave-on	Rinse-off
**NP + 4 NP**	y = 8721.0x - 257035	y = 6588.0x – 1E6	y = 8257.6x -2E6	0.8	0.9
**NPEO**	y = 341.8x - 6972	y = 341.7x -68686	y = 288.3x – 31344	1.0	0.8
**OPEO**	y = 365.0x - 26602	y = 562.5x + 27058	y = 352.7x + 24986	1.5	1.0
**NP40**	y = 974.7x + 137396	y= 763.4x - 19857	y = 985.5x + 196384	0.8	1.0
**TX-100**	y = 715.9x + 101445	y = 464.6x + 98288	y =708.3x + 21683	0.6	1.0

[a] SMM/SEC: Slope of the matrix-matched calibration between slope of the external calibration
